# Management of dyspepsia and *Helicobacter pylori* infection: the 2022 Indonesian Consensus Report

**DOI:** 10.1186/s13099-023-00551-2

**Published:** 2023-05-22

**Authors:** Ari Fahrial Syam, Muhammad Miftahussurur, Dadang Makmun, Murdani Abdullah, Abdul Aziz Rani, Gontar Alamsyah Siregar, Marcellus Simadibrata, Nasrul Zubir, I. Dewa Nyoman Wibawa, Hery Djagat Purnomo, Chudahman Manan, Dharmika Djojoningrat, Achmad Fauzi, Kaka Renaldi, Hasan Maulahela, Amanda Pitarini Utari, Rabbinu Rangga Pribadi, Virly Nanda Muzellina, Saskia Aziza Nursyirwan, Muhammad Firhat Idrus, Ruswhandi Ruswhandi, Titong Sugihartono, Muhammad Begawan Bestari, Putut Bayupurnama, Triyanta Yuli Pramana, Bogi Pratomo Wibowo, Achmad Fuad Bakry, Fardah Akil, Andi Muhammad Luthfi Parewangi, Haris Widita, I Ketut Mariadi, Ignatia Sinta Murti, Ali Imron Yusuf, Arles Arles, Fauzi Yusuf, Bradley Jimmy Waleleng, Abimanyu Abimanyu, Yustar Mulyadi, Maria Inge Lucida, Yudith Annisa Ayu Rezkhita, Ricky Indra Alfaray, Yoshio Yamaoka

**Affiliations:** 1 Division of Gastroenterology, Department of Internal Medicine, Faculty of Medicine-Cipto Mangunkusumo Teaching Hospital, University of Indonesia, Jakarta, Indonesia; 2 Division of Gastroentero-Hepatology, Department of Internal Medicine, Faculty of Medicine-Dr. Soetomo Teaching Hospital, Universitas Airlangga, Surabaya, Indonesia; 3 Helicobacter Pylori and Microbiota Study Group, Institute of Tropical Disease, Universitas Airlangga, Surabaya, Indonesia; 4 Division of Gastroenterohepatology, Department of Internal Medicine, Adam Malik General Hospital/Faculty of Medicine, Sumatra Utara University, Medan, Indonesia; 5 Division of Gastroenterohepatology, Department of Internal Medicine, M. Djamil General Hospital/Faculty of Medicine, Andalas University, Padang, Indonesia; 6 Division of Gastroenterohepatology, Department of Internal Medicine, Udayana University/Sanglah General Hospital, Bali, Denpasar, Indonesia; 7 Division of Gastroenterohepatology, Department of Internal Medicine, Kariadi General Hospital/Faculty of Medicine, Diponegoro University, Semarang, Indonesia; 8 Department of Internal Medicine, Gatot Subroto Army Central Hospital, Jakarta, Indonesia; 9 Division of Gastroenterohepatology, Department of Internal Medicine, Hasan Sadikin General Hospital/Faculty of Medicine, Padjadjaran University, Bandung, Indonesia; 10 Division of Gastroenterohepatology, Department of Internal Medicine, Sardjito General Hospital/Faculty of Medicine, Public Health and Nursing, Gadjah Mada University, Yogyakarta, Indonesia; 11 Division of Gastroenterohepatology, Department of Internal Medicine, Moewardi General Hospital/Faculty of Medicine, Sebelas Maret University, Surakarta, Indonesia; 12 Division of Gastroenterohepatology, Department of Internal Medicine, Saiful Anwar General Hospital/Faculty of Medicine, Brawijaya University, Malang, Indonesia; 13 Division of Gastroenterohepatology, Department of Internal Medicine, Moch. Hoesin General Hospital/Faculty of Medicine, Sriwijaya University, Palembang, Indonesia; 14 Division of Gastroenterohepatology, Department of Internal Medicine, Wahidin Sudirohusodo General Hospital/Faculty of Medicine, Hasanuddin University, Makassar, Indonesia; 15 Department of Internal Medicine, West Nusa Tenggara General Hospital, Mataram, Indonesia; 16 Department of Internal Medicine, Abdul Wahab Sjahranie General Hospital/Faculty of Medicine, Mulawarman University, Samarinda, Indonesia; 17 Department of Internal Medicine, Abdoel Moeloek General Hospital/Faculty of Medicine, Lampung University, Lampung, Indonesia; 18 Department of Internal Medicine, Awal Bros Pekanbaru Hospital, Pekanbaru, Indonesia; 19 Division of Gastroenterohepatology, Department of Internal Medicine, Zainoel Abidin General Hospital/Faculty of Medicine, Syiah Kuala University, Banda Aceh, Indonesia; 20 Division of Gastroenterohepatology, Department of Internal Medicine, Prof. R. D. Kandou General Hospital/Faculty of Medicine, Sam Ratulangi University, Manado, Indonesia; 21 Department of Internal Medicine, Ulin Banjarmasin General Hospital, Faculty of Medicine, Lambung Mangkurat University, Banjarmasin, Indonesia; 22 Department of Internal Medicine, Soedarso General Hospital, Pontianak, Indonesia; 23 Institute of Tropical Disease, Universitas Airlangga, Surabaya, Indonesia; 24 Department of Internal Medicine, Faculty of Medicine, Universitas Muhammadiyah Surabaya, Surabaya, Indonesia; 25 Department of Environmental and Preventive Medicine, Faculty of Medicine, Oita University, Oita, Japan; 26 The Research Center for GLOBAL and LOCAL Infectious Diseases (RCGLID), Oita University, Oita, Japan; 27 Department of Medicine, Gastroenterology and Hepatology Section, Baylor College of Medicine, Houston, USA

**Keywords:** Management, Dyspepsia, *Helicobacter pylori* infection, Indonesia, Human and illness

## Abstract

Dyspepsia still becomes a major challenge in upper gastrointestinal disease in Indonesia. This disease often correlated with *Helicobacter pylori* infection. However, the prevalence of this bacterium is generally low in Indonesia. Therefore, several considerations should be taken into consideration during the management of dyspepsia and *H. pylori* infection. “Management of dyspepsia and *H. pylori* infection in Indonesia: The Indonesian consensus report” comprises information gathered from 22 gastroenterology centers across Indonesia. The experts gathered to evolve a consensus, that consists of the statements, grades of recommendations, evidence levels, and rationales for the dyspepsia and *H. pylori* infection management for daily clinical practice. The report explains several aspects from the updated epidemiology information to comprehensive management therapy. After the experts worked together on all statements in the recommendations, the results are presented with the final agreement as a consensus to help clinicians in understanding, diagnosing, and treating dyspepsia and *H. pylori* infection patients in daily clinical practice in Indonesia.

## Introduction

Dyspepsia becomes the fifth and sixth most common disorder among inpatients and outpatients in Indonesia, respectively [1, 2]. This condition often associated with the infection of *Helicobacter pylori*, a stomach pathogen that causes gastrointestinal (GI) diseases including gastritis, gastric B-cell lymphoma, gastroduodenal peptic ulcer, and gastric adenocarcinoma [3]. While the prevalence of *H. pylori* infection in the neighbors and majority of Asian countries is high, Indonesia becomes unique since the infection rate this bacterium in the general population is low. Unfortunately, the incidence of dyspepsia remains high in the general population regardless of the prevalence of *H. pylori* infection. Therefore, a different approach is needed to effectively treat dyspepsia and *H. pylori* infection in Indonesia.

“Management of dyspepsia and *H. pylori* Infection: The Indonesian Consensus Report” gathered key opinion experts from across the country to review and assess clinical aspects of dyspepsia and *H. pylori* infection. Twenty-eight gastroenterologists and clinicians had a discussion in an integrated meeting and developed consensus statements, grades of recommendations, levels of evidence, and rationales for the dyspepsia and *H. pylori* infection management in daily practice in Indonesia. In addition, this consensus guideline contains a special topic on the dyspepsia management in patients with COVID-19. This renewed consensus for Indonesia was developed to summarize the current theory and perspectives on dyspepsia and *H. pylori* infection from several guidelines [1, 4–11] and subsequently adapted to the healthcare center conditions in Indonesia. This consensus referring ‘clinicians’ for the medical doctors while ‘health practitioner’ for all the health workers including medical doctors and nurses. In the future, at a minimum frequency of once every 5 years, this consensus report will be updated as the knowledge and understanding of dyspepsia and *H. pylori* infection increases.

## Methods

**Fig. 1:**
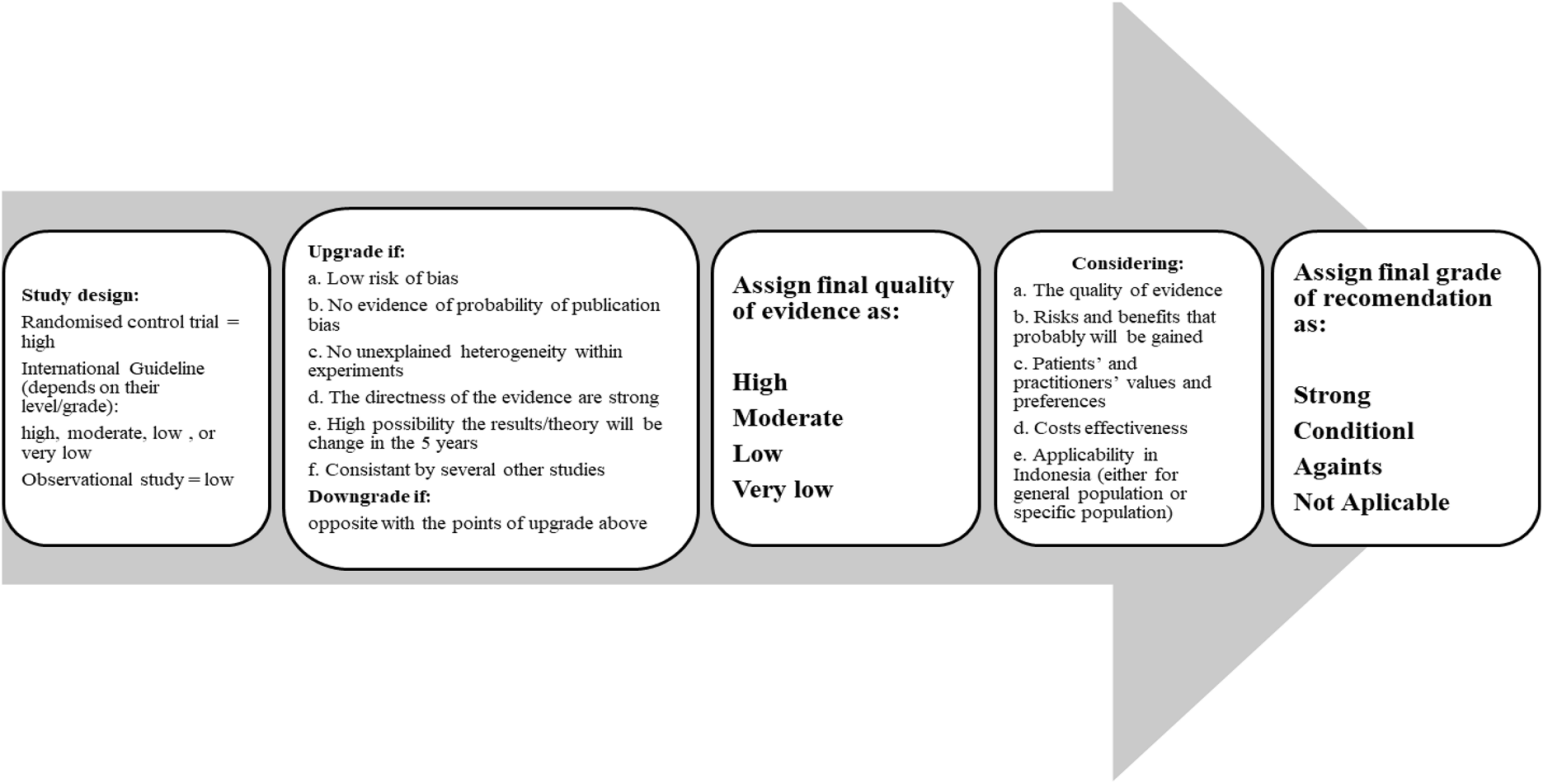
Steps for assigning the quality of evidence until grade of recommendation

The current knowledge, clinical practice evidence, published guidelines, and journals were collected, investigated, and analyzed by working groups in the workshop. The working group on each sub-topic constructed statements and rationale based on their expertise, and prepared a draft. Midway through the meeting, the working group leader, accompanied by the working group secretary, led the discussion on each statement. Subsequently, the statements were presented to all the key experts present and discussed to meet the standard template. Evidence quality is an objective and reproducible parameter that considers risk of study bias, evidence of possible publication bias, presence of unexplained heterogeneity within experiments, directness of evidence, and accuracy of estimates was evaluated during 2021 until 2022 period (Table 1; Fig. 1).

**Table 1:** Quality of evidence

Quality of evidence	Comments
High (level 1)	Estimation of effect by further research is very unlikely to change our confidence
Moderate (level 2)	Estimation of effect by further research is unlikely to change our confidence
Low (level 3)	Estimation of effect by further research is likely to have an important impact and change our confidence
Very low (level 4)	Estimation of effect is considerably very uncertain

The grades of recommendation were developed considering the quality of evidence, risks and benefits, as well as the values and preferences of the patients and health practitioners. The grades of recommendation were also developed considering the cost effectiveness, applicability, and general condition of health care centers in Indonesia. The considerations were then discussed and decided into grades of recommendation upon agreement among the experts (Table 2; Fig. 1).

**Table 2:** Grade of recommendation

Grade of recommendation	Comments
Strong	Most of the patients should receive the recommended management of action as the statements mentioned
Conditional	Several patients may follow the recommended management of action; however, alternative options could be suitable for other patients, necessitating further discussion so that each patient can make a decision based on their individual condition
Against	Most of the patients should not receive the recommended management of action as the statements mentioned
Not applicable	The statement is not correlated with a management recommendation (i.e., definition and prevalence); therefore, it cannot be applicable

The quality of evidence and grades of recommendation were developed in several steps as shown in Fig. 1. All statements and rationales were discussed and agreed upon the meeting. A consensus was achieved when at least 80% of the participants agreed. The final list of statements, grades of recommendation, level of evidence, and rationale was written by the secretary, reviewed by the working group leaders, and summarized in this consensus report.

### Dyspepsia

**Fig. 2:**
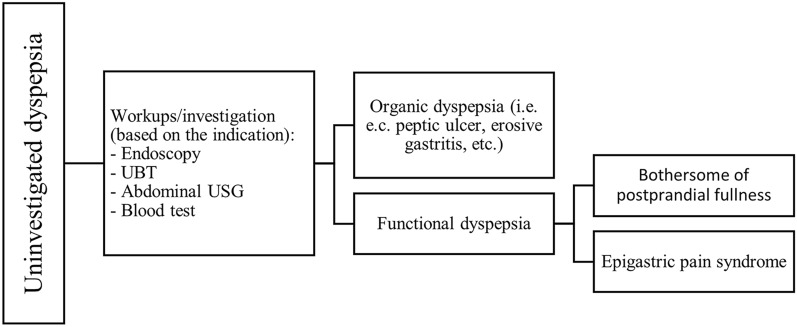
Algorithm for the diagnosis of uninvestigated dyspepsia

Dyspepsia often explained as chronic pain or disconcert localized to the upper abdomen [4, 12]. In this consensus, we described dyspepsia as any persistent discomfort feeling (e.g., epigastric pain, burning feeling, postprandial fullness, and early satiety) originating from the upper abdomen or GI tract. Dyspepsia can be classified as organic or functional dyspepsia (FD). Organic dyspepsia can be defined as dyspepsia that induced by known etiology that diagnosed after thorough investigation especially concerning structural disease (e.g., endoscopic lesion). The example etiology or risk factor of organic dyspepsia are duodenal or gastric ulcer, erosive gastritis, duodenitis, gastritis, and malignant processes. FD can be defined as dyspepsia with the absence of structural disease after the investigation using imaging, endoscopy, or similar method. The etiology of FD is most likely multifactorial with the exact cause remain unclear. The female sex, rise of age, high socioeconomic status, decreased of urbanization, infection of *H. pylori*, macro and micronutrient intake in dietary habits, and nonsteroidal anti-inflammatory drug use are risk factor for dyspepsia [1, 13–15]. In general, FD can be classified as Postprandial Distress Syndrome (PDS) and Epigastric Pain Syndrome (EPS) (Fig. 2) [1, 12]. PDS primarily involves early satiety or postprandial satiety, and EPS primarily includes epigastralgia or burning [16].

Although it is often benign, especially for FD, dyspepsia has been observed reduce the quality of life. A multi-center Asian study comprising 1115 patients with un-investigated dyspepsia from nine countries including Indonesia revealed that 43% of the patients were shown to have FD [17]. According to data from the Ministry of Health, Republic of Indonesia, dyspepsia was the fifth and sixth most prevalent disease in inpatients and outpatients in Indonesia [18]. The risk factors for patients with dyspepsia such as macronutrient and micronutrient intake in dietary habits in Indonesia may vary among sub-populations [19]. Therefore, determining a guideline for dyspepsia management in daily clinical practice is necessary.

#### Diagnosis and evaluation of patients with dyspepsia

##### Statement 1

The diagnosis of ‘FD’ can be made if the patients mentioned to have a syndrome; however, the upper GI endoscopy or imaging investigation do not show any structural abnormality that can explain the symptoms (unexplained after a routine clinical evaluation). The syndrome is a group of patient’s complaints with one or more the following symptoms, which have been present within the past 3 months in at least 6 months from the previous onset: epigastric pain, burning feeling, uncomfortably postprandial satiety, and early or quick satiety. The diagnosis of ‘organic dyspepsia’ can be made by clinicians if the patients mentioned the syndrome and the upper GI endoscopy or imaging investigation clearly show any structural abnormality that may explain the symptoms. The diagnosis of ‘un-investigated dyspepsia’ can be made if the patients experience any persistent discomfort feeling as likely to be the dyspepsia syndrome in the upper abdomen; however, the upper GI endoscopy or imaging investigation has yet to be done.

**Grade of recommendation:** Strong.

**Level of evidence:** High.

**Rationale:**

Dyspepsia described as chronic pain or disconcert localized to the upper abdomen [4, 12]. Patient with single or multiple symptoms related with gastroduodenal abnormalities (e.g., epigastric pain and burning, postprandial satiation, early satiety, etc.) according to Rome III and IV criteria Indigestion is diagnosed. However, these criteria remain somewhat vague and can be difficult to interpret for patients and physicians. The British Gastroenterology Society defines dyspepsia as a group of upper gastrointestinal symptoms lasting more than 4 weeks [20]. Dyspepsia due to structural abnormalities or another specific etiology can be classified as organic dyspepsia while dyspepsia with unclear etiology can be likely classified as FD [21].

FD describe as disease with one or more gastroduodenal manifestations based on Rome IV criteria. The signs included postprandial satiety, early satiety, sensation of epigastric pain and burning, with no evidence of structural disease (including upper endoscopy). According Rome III criteria, a diagnosis of FD can be made without requiring a minimum frequency of occurrence. Criteria are met if symptoms persist for at least 3 months in his 6 months prior to diagnosis. Likely no signs of structural disease to explain symptoms [12, 22–25]. Of note, multiple organic, systemic, or metabolic disorders of and medications that can cause symptoms resembling organic dyspepsia and should be considered withdrawn from FD diagnosis. The differential diagnosis of FD includes for example: gastritis, peptic ulcer disease (PUD), GI and hepatobiliary cancers, parasitic infections, *H. pylori* infections, celiac disease, gastroparesis, small intestinal bacterial overgrowth, irritable bowel syndrome, chronic pancreatic disorders, hyper- and hypothyroidism, acute cholecystitis, chronic renal failure, electrolyte imbalances, and medications [17, 21].

##### Statement 2

The alarm symptoms of dyspepsia are still beneficial in Indonesia. Thus, health practitioners should understand and apply observation of the alarm symptoms of dyspepsia during clinical practice. Patients with the alarm symptoms should prompt referral for the upper endoscopy investigation.

**Grade of recommendation:** Strong.

**Level of evidence:** High.

**Rationale:**

The alarm symptoms of dyspepsia involve weight loss (unintended weight loss), continuous dysphagia, constant vomiting, gastrointestinal bleeding, anemia, fever, mass in the upper abdomen, family history of stomach cancer, and age 50 years [1, 26]. As many as 13% and 4% of patients with alarm symptoms who underwent endoscopy were diagnosed with clinically significant peptic ulcer disease and gastric cancer, respectively [26]. Patients with the alarm symptoms in Indonesia may not because of *H. pylori* infection since the prevalence of this bacterium infection is low in general population. This condition may lead to more serious differential diagnosis during the etiology analysis. Therefore, even though there only 313 hospitals in Indonesia have gastrointestinal endoscopy systems with most of them in mainland Java [18], patients with the alarm symptoms should referred to the health care centers (hospital) where the endoscopy investigation could be performed [26, 27]. Careful observation and management should be performed by monitoring the patients’ health condition according to the patient’s and health care centers’ situation. The health practitioners should be wary of the new onset of dyspepsia and alarm symptoms in the above patients [1].

##### Statement 3

Endoscopy investigation is suggested to exclude upper GI neoplasia or other organic diseases in dyspeptic patients with aged 50 years or greater, dyspepsia patients with the alarm symptoms, and/or patients presenting with symptoms that are non-responsive to the initial treatment.

**Grade of recommendation:** Conditional.

**Level of evidence:** Moderate.

**Rationale:**

Gastric cancer is the fifth highest incidence among cancers worldwide and as the fourth most prevalent cause of cancer-related death globally (1) and frequently presents with dyspepsia. In Indonesia, the new case and risk of gastric cancer is low (19th rank in new case of cancer) (2); however, some ethnic groups had severe gastric mucosal disease as a hallmark of high-risk populations (3). Endoscopy is not widely available in all areas in Indonesia; thus, stratifying the risk by the alarm symptoms is necessary to increase the cancer detection rate (4).

#### Management of dyspepsia

##### Statement 4

Patients with dyspepsia should undergo initial treatment with empirical proton pump inhibitor (PPI) therapy with or without a prokinetic if there is no alarm symptom.

**Grade of recommendation:** Strong.

**Level of evidence:** High.

**Rationale:**

**Fig. 3:**
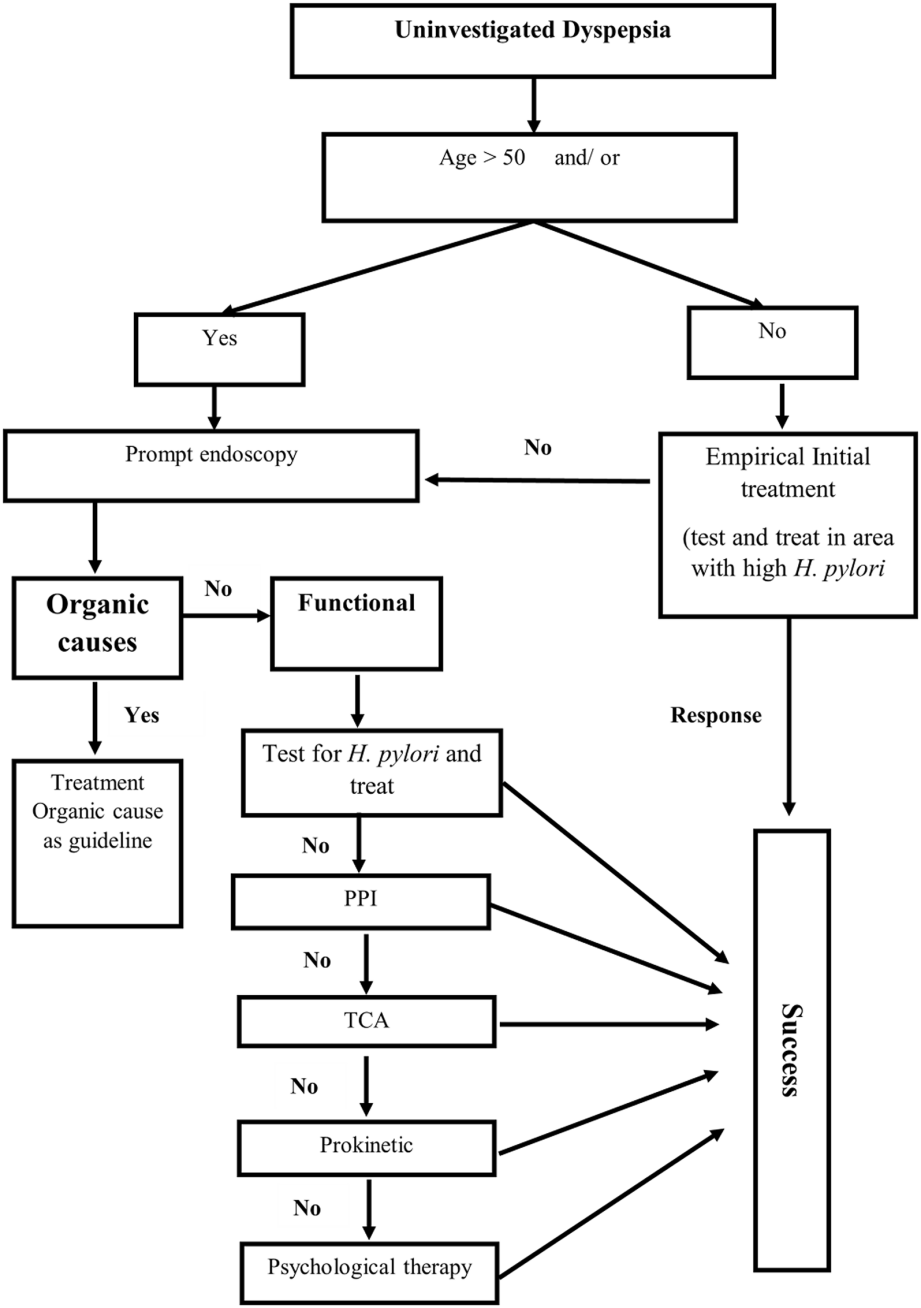
Algorithm for the management of dyspepsia

PPI therapy is superior to placebo or antacid therapy in treating dyspepsia. The test and treat strategy may be cost-effective when applied to the regions in Indonesia with high prevalence of *H. pylori* infection (please refer to *H. pylori* infection consensus section). Exercise-promoting therapy should be used with caution and at the lowest effective dose (e.g., metoclopramide for < 12 weeks, domperidone doses ≤ 30 mg daily) (Fig. 3) [4, 6].

##### Statement 5

After *H. pylori* eradication, FD patients with any dyspepsia symptom should be treated with a PPI.

**Grade of recommendation:** Conditional.

**Level of evidence:** Medium.

**Rationale:**

Symptoms that might appear after *H. pylori* eradication may vary among heartburn, epigastric pain, nausea, or other symptoms [28]. PPI therapy has a statistically significant impact on dyspepsia symptoms with a number needed to treat (NNT) of 10 (95% CI: 7–20). Overall, 69.6% patients of PPI group had persistent dyspeptic symptoms in comparison with 75.2% control group [6]. However, if PPI therapy is observed to no longer beneficial, it should be stopped and evaluated [4].

##### Statement 6

Tricyclic antidepressants (TCAs) and prokinetics can be considered as an optional therapy for patients with FD in whom treatment with PPIs failed. An evaluation or re-evaluation of *H. pylori* infection status should be conducted for such patients.

**Grade of recommendation:** Conditional.

**Level of evidence:** Moderate.

**Rationale:**

Based on the excellent evidence for TCAs in this indication, TCAs should be administered before prokinetic drugs to treat FD. TCAs have been shown to be highly effective in treating patients with FD [29]. TCAs are commonly associated with adverse events (constipation, dry mouth, urinary retention, and somnolence) [6, 10, 29]. Evaluation or re-evaluation of the *H. pylori* infection status should be conducted as there is still a possibility of recurrence or recrudescence even after eradication therapy, which can cause the symptoms to persist [26, 30]. In addition, a recent meta-analysis study showed that TCAs, but not Selective Serotonin Reuptake Inhibitors (SSRIs), are efficacious in the treatment of FD, but antidepressants were also associated with a higher incidence of adverse events than placebo [29].

##### Statement 7

Psychological therapies should be considered for FD patients with no response prior to drug therapy.

**Grade of recommendation:** Conditional.

**Level of evidence:** Low.

**Rationale:**

A review that involved a total of 12 Randomized Controlled Trials (RCT) in FD patients showed that psychological therapies will give significant benefit over the control group. Studies suggested that psychological therapies have a significant benefit of reducing dyspepsia symptoms (RR = 0.53; 95% CI: 0.44–0.65) with an NNT of 3 (95% CI: 3–4). The most common approaches included cognitive behavioral therapy or other various forms of psychotherapy. Although a dramatic effect was observed with regard to the reduction of dyspepsia symptoms, the quality of the data is very low [6]. Future RCT-based study in Indonesia is needed to provide more evidence of psychological therapies benefits towards FD patients.

#### Dyspepsia patients with COVID-19

##### Statement 8

Patients’ dyspepsia with COVID-19 should be carefully evaluated whether the etiology of dyspepsia is because of the viral infection or there is another etiology.

**Grade of recommendation:** Conditional.

**Level of evidence:** High.

**Rationale:**

COVID-19 can have GI manifestations, including symptoms related to dyspepsia, during or post the disease process. The symptoms such as nausea and mild and transient vomiting might cause by a gastrointestinal response to the severe acute respiratory syndrome coronavirus 2 (SARS-CoV-2) infection or to antiviral medication [31]. Some of the GI manifestations could also be a predictor of worse prognoses of COVID-19 [26]. Specific to the upper GI, this might be due to direct pathological pathways since viral nucleocapsid proteins were detected in the cytoplasm of the stomach cells [32–34]. In Indonesia, several studies reported that dyspepsia may occur during or post COVID-19 infection [35, 36]. Not only dyspepsia but other severe conditions induced by organic dyspepsia might also occur. For example, in other countries, it was reported that approximately 4% of patients with SARS-CoV-2 pneumonia had gastrointestinal bleeding [37]. Thus, the etiology of dyspepsia in patients with COVID-19 should be evaluated carefully. The evaluation should begin with the profound anamnesis to dispose any other possible differential diagnosis.

##### Statement 9

When the onset of dyspepsia occurs likely together with the first onset of COVID-19 symptoms and most of differential diagnosis for ‘organic dyspepsia’ can be eliminated, the clinicians should consider the diagnosis as ‘organic dyspepsia et causa COVID-19’.

**Grade of recommendation:** Strong.

**Level of evidence:** High.

**Rationale:**

Generally, the diagnosis of dyspepsia in COVID-19 patients is the same as the diagnosis of dyspepsia in general (Statement 1). There is no clear difference in treatment or diagnosis of dyspepsia between patients who test positive or negative for COVID-19. Diagnosis and management should be done with caution, but as a mandatory standard he should ensure certain protection through the use of PPE [38]. The clinical and procedural guidelines provided by the experts should be implemented thoroughly, especially while carrying out invasive management such as endoscopy [39]. The health practitioners should use personal protective equipment (PPE) to prevent infection with the virus.

##### Statement 10

The clinicians should carefully determine the management therapy for dyspepsia patients with COVID-19 in order to get the best therapeutic option with less side effect to the upper GI tract.

**Grade of recommendation:** Conditional.

**Level of evidence:** Low.

**Rationale:**

Management of patients with dyspepsia and COVID-19 is the same as management of patients with systemic dyspepsia (Statement 4). Several risk factors lead to damage to the gastric mucosa from stress in COVID-19 patients, especially in critically ill patients. These include mechanical ventilation, hypoxia, multiple organ failure, psychological stress, and acute respiratory distress syndrome. Theoretically, the COVID-19 patients, especially during their critical condition, should have a higher incidence of stress-induced gastric mucosal damage. PPIs can be used as an option to prevent stress-induced gastritis erosion in COVID-19 patients with such risk factors. Additionally, enteral nutrition and mucosal protectants help protect the gastrointestinal mucosa. Other recommended treatments, such as antipyretic, liver support, management of drug-related adverse events, and psychotherapeutic support, may also be provided as needed. Metoclopramide, domperidone, or 5-hydroxytryptamine receptor antagonists are preferred treatment options for nausea and vomiting [31].

Next, the management therapy (e.g., antiviral and vitamin including their dose) should be chosen carefully in order to get the best option with less side effect to the upper GI tract. The 4th Edition of Indonesian COVID-19 Management Guideline (published in 2022) stated that antiviral drugs including Favipiravir, Redesivir, Molnupiravir, and Nirmatrelvir/Ritonavir with several doses’ regimen can be used as the treatment for COVID-19 infection [40]. According to The Indonesian Food and Drug Authority and previous studies, while all these drugs potentially induce upper GI tract symptoms such as nausea, vomiting, and abdominal pain, certain drug reported to have lower side effect compared to other [41–43]. Single drug regimen and lower dose regimen are desirable to reduce the risk of nausea, vomiting, and abdominal pain. Combination between drugs should be assessed carefully. For example, previous studies showed that GI adverse events were more commonly found in patients with LPV/r (a Lopinavir-Ritonavir recombinant therapy) compared to any other regimen therapy. Compared to LPV/r, single regimen of Favipiravir showed a lower side effect of nausea, vomiting, and abdominal pain, thus it might better to use Favipiravir only than LPV/r in dyspepsia patients if there is no special reason to use LPV/r regimen [41]. Further study needs to be governed in order to understand best regimen option for COVID-19 patient with dyspepsia in Indonesia.

#### *Helicobacter pylori* infection

The *H. pylori* infection rate in Indonesia is low compared to other Asian countries [44, 45]. A preliminary study showed that of 267 patients have symptoms of dyspepsia from the five largest islands in Indonesia, 22.1% (59/267) of patients were positive for *H. pylori* infection based on the criteria of a minimum of one positive test result from the four diagnostic test methods: culture, histological, immunohistochemistry (IHC), and rapid urease test (CLO test, Kimberly-Clark, USA) [46]. Furthermore, a prospective study including 1053 patients from 19 cities across Sumatra, Java, Borneo, Bali, Sulawesi, Timor, and Papua Island confirmed this low prevalence (10.1%) in the general populations, even though some populations tend to have higher prevalence compared to others [47]. Source of drinking water, age, and religion, were risk factors for *H. pylori* infection; however, only ethnicity could be considered as an independent risk factor for *H. pylori* infection in Indonesia [48]. Future studies on a larger study population are needed to achieve an accurate representative number of the Indonesian population. Nevertheless, since different islands and cities have different prevalence of *H. pylori* infection (Table 3), management consensus of *H. pylori* infections remains desirable.

**Table 3:** Prevalence of *H. pylori* infection in Indonesia [46, 55, 81]

Island (city)	*H. pylori* prevalence
Bali (Bangli)	12%
Java	2.4–4%
(Surabaya)	5%
(Jakarta)	0.03%
(Malang)	1%
(Semarang)	0%
Kalimantan (Pontianak)	6.7–7.5%
Papua (Jayapura)	43%
Sumatera	20%
(Medan)	27.9–40%
(Aceh)	0%
Sulawesi	15%
(Manado)	12%
(Makassar)	20%
Timor (Kupang)	36.7–40%

#### Epidemiology and disease-related *H. pylori*

##### Statement 11

Improvement of sanitary and hygiene conditions (e.g., source of drinking water) is important and need to be governed to minimize the prevalence of *H. pylori* in Indonesia. The knowledge regarding sanitary and hygiene should be propagating to every elements of communities as part of main health promotion programs especially by primary health care units.

**Grade of recommendation:** Strong.

**Level of evidence:** High.

**Rationale:**

Sanitary and hygienic conditions especially the drinking water sources are known risk factors for *H. pylori* infection and are associated with poor household hygiene when contracting this infection. A study in Indonesia, where data were adjusted for age and sex, found that people who used tap water as their drinking water source had significantly lower infections than those who drew water from a well/river [46, 49, 50].

##### Statement 12

*H. pylori* infection is still a risk factor for dyspepsia and other gastroduodenal diseases including in the low infection prevalence area.

**Grade of recommendation:** Not applicable.

**Level of evidence:** High.

**Rationale:**

*H. pylori* infection was shown to be more common in patients with dyspepsia than in asymptomatic controls or patients with gastric ulcer, gastric cancer, and duodenal ulcer. Disease symptoms reflect the pattern and degree of gastritis or gastric atrophy. Even in areas with low prevalence of *H. pylori* infection, the clinicians still can find patients with *H. pylori*-positive (e.g. Surabaya where the Chinese ethnicity tend to have positive results of *H. pylori* infection compared to Javanese) [51]. *H. pylori* positive patients showed more severe disease compared with *H. pylori*-negative patients by histopathological examination [51]. Thus, regardless of where the patients were from, if the patients have *H. pylori* infection, eradication therapy must be initiated. In addition, other factors such as diet/nutrition pattern need to be monitored since they have an effect on dyspepsia and other gastroduodenal diseases [1, 7, 15, 52].

##### Statement 13

The low gastric cancer incidence in Indonesia not only due to low *H. pylori* infection prevalence.

**Grade of recommendation:** Not applicable.

**Level of evidence:** High.

**Rationale:**

The risk of gastric cancer in Indonesia is low supported by the low intestinal metaplasia grade in Indonesian general population [53]. Even within *H. pylori* infected patients, recent study revealed that the proinflammatory cell infiltration and cytokine expression response in Indonesian population during *H. pylori* infection is generally not robust, thus, reducing the risk factors of gastric cancer development [53]. Another possible reasons could be that sodium consumption among the Indonesian population was generally lower than that in other countries, especially other than South-East Asian countries [54–56].

While Indonesian population generally have low gastric cancer incidence, however, patients from Timor, Papua, and Bugis ethnic groups remain showed higher gastric cancer risk factors compared to other ethnicities [47]. Even without *H. pylori* infection, these ethnicities tend to have higher pro-inflammatory cytokines expression compared to others [53]. Thus, special consideration must be given if the patients come from these ethnicities. In addition to ethnicity, factors such as diet (overconsumption of sodium, fat, and retinol), atrophic gastritis, family history of gastric cancer, possibly higher body mass index, and decreased serum HDL (high-density lipoprotein) levels are another risk factors of gastric cancer that should be monitored [57, 58].

##### Statement 14

Current evidence primarily supports extra-intestinal manifestations of *H. pylori* in immune thrombocytopenic purpura (ITP), iron deficiency anemia (IDA), urticaria, Parkinson's disease, migraine, and rosacea. However, the scientific evidence of relationship between *H. pylori* infection with other diseases remain warrant further in-depth study. Studies with these topics need to be conducted in Indonesia especially in area with high prevalence of *H. pylori* infection.

**Grade of recommendation:** Not applicable.

**Level of evidence:** Moderate.

**Rationale:**

*H. pylori* infection is associated with many diseases. However, the causality of these associations has not yet been confirmed in Indonesia. These include hematological, cardiopulmonary, cardiovascular, metabolic, nervous, and cutaneous systems (chronic urticaria, rosacea), and autoimmune diseases (e.g., Sjögren's syndrome, hypothyroidism, and Henoch-Schönlein purpura) [6, 10, 59, 60]. A study also confirmed that the protective hypothesis for asthma in populations with poor sanitation and low *H. pylori* prevalence did not confirm a protective effect [61]. The status of *H. pylori* infection and chronic urticaria had no correlation [62]. Further in-depth study needs to be governed especially in area with high prevalence of *H. pylori* infection in Indonesia.

#### Screening the infection and disease-associated *H. pylori*

##### Statement 15

Serum pepsinogen (PG) and *H. pylori* antibody testing for community screening should be used in area with high prevalence of *H. pylori* infection. This screening method should be used with caution in areas with low prevalence of *H. pylori* infection.

**Grade of recommendation:** Strong.

**Level of evidence:** Strong.

**Rationale:**

A combination of serum PG and *H. pylori* antibody test called the ABC method is a recommended to be used for community screening in many countries including Indonesia [63]. Previous study showed that this method is favored to be used especially in area with high prevalence of *H. pylori* infection in Indonesia. In areas with low prevalence of *H. pylori* (e.g., Java and Borneo/Kalimantan), the clinicians should be careful to interpret the result since patients with false-positive results may fall into group D, the highest risk group for stomach cancer [64]. In addition, special for PG, previous study in Indonesians found that this biomarker levels is useful to determine chronic gastritis in dyspepsia patients [64, 65].

##### Statement 16

Screening for gastric cancer should be implemented, particularly in areas with a high prevalence of *H. pylori.*

**Grade of recommendation:** Conditional.

**Level of evidence:** High.

**Rationale:**

In Indonesia, general prevalence of *H. pylori* is low which is similar to the intestinal metaplasia and gastric cancer prevalence [53]. However, ethnicities such as Timor, Papuan, Batak, and Bugis were proven to have higher risk of *H. pylori* infection and gastric cancer, regardless of where they live. In the area with *H. pylori* infection and gastric cancer are higher than that in the general population, screening via the “test-and-treat strategy” should be implemented [46, 66].

#### *H. pylori* infection management

##### Statement 17

A test-and-treat strategy is appropriate for un-investigated dyspepsia in Indonesia, especially in areas of with intermediate to high level of *H. pylori* infection prevalence. However, this strategy should not be implemented to the patients with the alarm symptoms or older patients more than 50 years old.

**Grade of recommendation:** Strong.

**Level of evidence:** High.

**Rationale:**

Patients under 50 years with symptoms of dyspepsia and no warning signs was recommended to A “test and treat” experience. This strategy prioritizes noninvasive testing over prescription PPI or direct esophagogastroduodenoscopy (EGD) to avoid cost, inconvenience, and discomfort. Although there were no group differences in symptom resolution at 12 months, the group assigned to testing and treatment will give lower overall costs [4, 6].

A “test-and-treat” strategy for *H. pylori* was shown to be more effective than direct symptomatic therapy for dyspepsia patients without *H. pylori* test (relative risk 0.59; 95% CI:0.42–0.83). The prevalence of infection modifies the predictive value of diagnostic methods. In the low prevalence of *H. pylori* infection populations, test-and-treat strategy should be used with caution when the clinicians choose to use an invasive method [4, 67, 68].

##### Statement 18

Diagnostic tests for *H. pylori* infection include the following: locally validated urea breath test (UBT), stool antigen test (SAT), immunological test (urine and serum serology), rapid urease test (RUT), histology, IHC, and culture. The diagnostic modalities recommended in Indonesia are UBT, RUT, and histology. To date, SAT does not show a high accuracy results in Indonesia. Urine and serum serology should not be used to determine recent infections of *H. pylori* infection.

**Grade of recommendation:** Strong.

**Level of evidence:** High.

**Rationale:**

Diagnostic tests of *H. pylori* infection can be performed both non-invasively/indirectly (urea breath test, stool antigen test, and antibody-based test, including serology and urine test) and invasively/directly using endoscopy (Table 4). The urea breath test is the most popular and convenient non-invasive tests with a diagnostic accuracy > 95%. Monoclonal enzyme immunoassay stool antigen tests also offer high sensitivity and specificity, greater than 95%, but the fecal gather is often associated with patient reluctance [1, 69, 70]. *Campylobacter-*Like Organism (CLO) Rapid Urease Test detects *H. pylori* infection by placing a biopsy specimen in a solution of urea and a pH-sensitive dye. The CLO test has a sensitivity of over 90% and a specificity of over 95%. Histology is possible using staining with hematoxylin and eosin or Giemsa. Culturing bacteria from biopsy specimens enables antimicrobial susceptibility testing [71–73].

**Table 4:** *H. pylori* diagnostic tests and the current situation in Indonesia [64, 71, 72, 92–94]

Diagnostic test	Sensitivity (%)	Specificity (%)	Advantage	Disadvantage	Situation in Indonesia	Refs.
Non-invasive test						
UBT	95	95	High accuracy Detects current infection	Less reliable in patients with a history of gastric resection or PPI consumption	13C-UBT and 14C-UBT remain restricted to 4 and 6 cities, respectively Expensive and uncovered by social insurance Ongoing validation	[54]
SAT	66.7–94	78.9–92	Inexpensive and not age dependent Novel monoclonal antibodies are not influenced by PPIs ICA-based, does not require special equipment or experts	Inconsistent accuracy based on antigens Accuracy influenced by incubation time and stool condition	Most centers use ICA-based tests, but with low sensitivity Collecting stool samples is more difficult than collecting blood samples	[48, 54]
Serology	66.7–90	80–97.2	Saves costs and reduces the endoscopic workload	Less accurate in children Wide range of cutoff values Cannot distinguish between current and past infections Lower accuracy than ICA-based tests	Validated for some kits. Should not be used solely to diagnose *H. pylori* infection	[40, 54]
Urine test	83	95	Easy sampling method without the need for special skills and tools Sampling is cheaper than serum sampling	False negative results with low concentrations of IgG	Lower accuracy Requires more time for interpretation; Lack of availability. Should not be used to diagnose *H. pylori* infection	[50]
Invasive test						
RUT	90	95	Rapid result warranting the fast management for *H. pylori* eradication	False negative in patients with recent GI bleeding or with the use of PPIs, antibiotics, or bismuth containing compounds	Validated for some kits	[53]
Histology	42–99	100	Histochemical staging is the standard for *H. pylori* gastritis assessment Widely available	In cases with low levels of *H. pylori*, histological stains can provide a negative result	Widely available in Indonesia	[51]
IHC	65–98	100	IHC staining for *H. pylori* has a lower inter-observer variation compared to histo- chemical stains	IHC staining procedure is more expensive than histochemical stains and it is not available in all laboratories	In cases of chronic (active) gastritis in which *H. pylori* is not detected by histochemistry, IHC of *H. pylori* can be used as an ancillary test	[51]
Culture	55–73	100	Allows an evaluation of antibiotic resistance irrespective of the intrinsic mechanism involved	*H. pylori* is difficult to culture	Only available in some centers	[49, 52]

##### Statement 19

Several conditions have been reported to be closely associated with or affected by *H. pylori* infection. Individuals with the following conditions should be considered for *H. pylori* testing:

- Peptic ulcer disease, either active or previous
- Gastric mucosa-associated lymphoid tissue (MALT) lymphoma
- Gastric cancer
- Long term aspirin and NSAID use
- Unexplained iron deficiency anemia (IDA)
- Idiopathic thrombocytopenic purpura (ITP)
- Functional dyspepsia
- GERD patients requiring long term PPI therapy

**Grade of recommendation:** Conditional.

**Level of evidence:** Moderate.

**Rationale:**

Patients with active PUD, a history of PUD, low-grade gastric mucosa-associated lymphoid tissue (MALT) lymphoma, or previous endoscopic resection of early gastric cancer (EGC) should be tested for *H. pylori* infection. Testing might minimize the risk of ulcer bleeding may be considered in patients taking low-dose aspirin for a long time. Eradication therapy should be suggested to positive patients. ITP in adulthood and her IDA of unknown cause should also be tested [4, 74].

In patients with GERD, bacterial testing is necessary, especially if clinical symptoms that require testing for *H. pylori* are found*.* The Asia–Pacific Consensus also propose eradication of *H. pylori* in patients requiring long-term her PPI [7].

##### Statement 20

If the *H. pylori* test result is positive, the bacteria must be eradicated.

**Grade of recommendation:** Strong.

**Level of evidence:** High.

**Rationale:**

Eliminating infection diminishes the risk of developing atrophic gastritis and gastric cancer. Although the low incidence of gastric cancer in Indonesia is due to low *H. pylori* infection, early diagnosis and treatment of symptomatic patients is necessary to decrease the risk of chronic complications [51, 73, 75].

##### Statement 21

When the indication for endoscopy is found, endoscopy should be performed, and the biopsy samples should be collected according on the Updated Sydney System guideline recommendation. Collecting biopsy sample for *H. pylori* diagnosis must be performed especially in the area with high prevalence of *H. pylori* infection or area with high risk of gastric cancer.

**Grade of recommendation:** Strong.

**Level of evidence:** High.

**Rationale:**

Endoscopy was indicated in several cases including for the patients with dyspepsia with concomitant alarm symptoms like weight loss, persistent vomiting, gastrointestinal bleeding, mass or IDA [57]. Biopsy should be taken carefully to get optimum results of histological examination. Several considerations such as the observation of baseline is important because when the pre-cancerous lesions at baseline found, the patients will be more likely to develop gastric cancer [73].

**Fig. 4:**
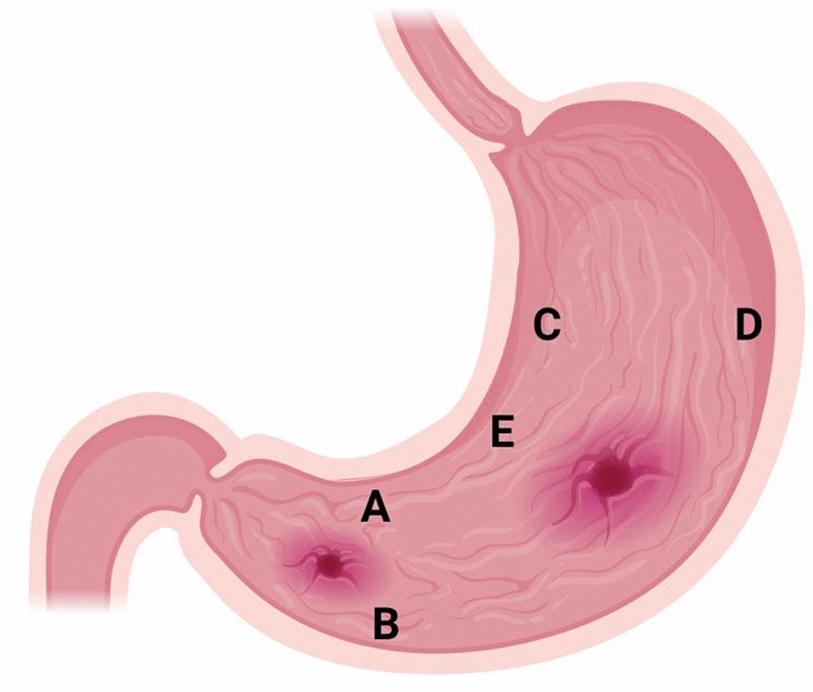
Locations of gastric biopsy recommended by the updated Sydney System. **a** Lesser curvature of the antrum; **b** greater curvature of the antrum; **c** lesser curvature of the body; **d** greater curvature of the body; and **e** incisura angularis [2]. Avoid taking biopsy specimens directly from the ulcer site

The ideal biopsy sites for the rapid urease test that recommended by the Updated Sydney System are the corpus and incisura regions [76] (Fig. 4). It is important to obtain biopsy samples from area with visible lesions such as area near the ulcers and suspicious focal lesions. Image-guided endoscopy accuracy in trained hands further increases targeted biopsy yield [11], thus endoscopist should improve their skill and updating their knowledge by attending training, workshop, seminar or similar clinical-academic events when it is possible.

##### Statement 22

The endoscopic diagnosis of *H. pylori* using conventional (white light endoscopy) and image-enhanced endoscopy for targeted biopsy can accurately detect the presence of *H. pylori* infection, after appropriate training.

**Grade of recommendation:** Conditional.

**Level of evidence:** Low.

**Rationale:**

It has long been suspected that the appearance of the gastric mucosa changes after *H. pylori* infection is unique, thus, provides useful diagnostic information for endoscopists. The Kyoto Classification of Endoscopic Gastritis was published in Japan in 2014, enabling diagnosis of *H. pylori* gastritis and assessment of gastric cancer risk under endoscopy [77, 78].

Atrophy, intestinal metaplasia, nodularity, and enlarged and tortuous folds have been described to be associated with the risk of gastric cancer [79]. Atrophic changes and intestinal metaplasia can be accurately identified using conventional image-guided endoscopy. However, proper assessment of the gastric mucosa to diagnose *H. pylori* infection by endoscopy requires proper training [11].

##### Statement 23

PPI therapy should be stopped approximately one to two weeks before testing for *H. pylori* infection; antibiotics (related to *H. pylori* eradication) and bismuth should be discontinued for 4 weeks before testing.

**Grade of recommendation:** Strong.

**Level of evidence:** High.

**Rationale:**

PPIs show anti-*H. pylori* activity and decrease the load of *H. pylori*, leading to false-negative results on urease test, UBT, and SAT. Two weeks is considered as a safe interval to avoid PPI use before testing for *H. pylori*, whereas a 1-week withdrawal has been shown to be sufficient [6]. In addition, antibiotics associated with eradication of *H. pylori* suppress infection and reduce test sensitivity and should be avoided 4 weeks prior to testing. Serological testing to detect antibodies to *H. pylori* infection is the only method that is unaffected by the use of PPIs, and use of the other diagnostic tests described above can lead to false-negative results [73, 80].

##### Statement 24

The first line eradication therapy of *H. pylori* used PPI triple therapy (PAC: PPIs, amoxicillin, and clarithromycin). The duration of therapies of 14 days. Nevertheless, this therapy should be implemented with caution in some regions in Indonesia with Clarithromycin resistant data higher than 10%.

**Grade of recommendation:** Strong.

**Level of evidence:** High.

**Rationale:**

**Fig. 5:**
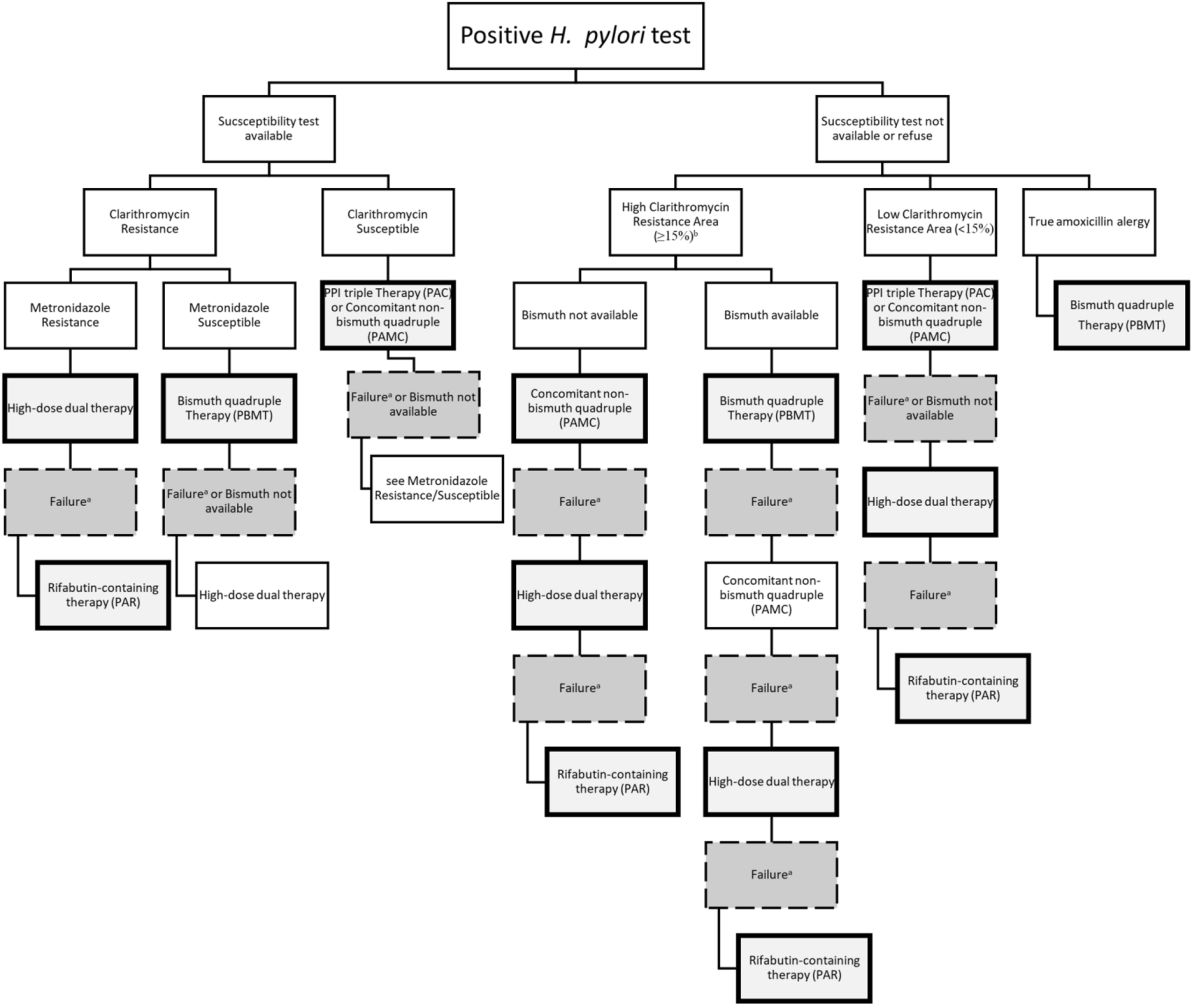
Algorithm of *H. pylori* antibiotic therapy. **a** In case of failure, an antibiotic susceptibility test is strongly recommended. The next antibiotic regimen should be chosen based on the results of the antibiotic susceptibility test. **b** Bali is an area with high clarithromycin resistance (≥ 15%). **c** Areas with high resistance to levofloxacin (≥ 15%) are Bali, Java, Kalimantan, Papua, Sulawesi, Sumatra, and Timor

A study in Indonesia revealed that the Indonesian population generally had a low prevalence of clarithromycin and amoxicillin resistance [81]. Thus, these drugs can be used as the first line of *H. pylori* eradication therapy in almost all rea of Indonesia. The therapy can be administered as PPI bid, amoxicillin 1000 mg bid, and clarithromycin 500 mg bid. Currently, only the population in Bali showed a resistance of clarithromycin > 15%. Thus, PPI triple therapy should not be used in Bali since may not be effective; therefore, it is better to use another regimen. The risk of 7-day treatment versus 14-day treatment failure in a given individual depends on the regional prevalence of antibiotic resistance, as 14-day treatment can overcome resistance to the antibiotics used. The balance between local failure rates and side effects should be determined based on locally validated data, as longer treatment regimens lead to longer durations of minor side effects [82] (see Table 5 and Fig. 5 for detailed information).

**Table 5:** Recommended lineage regimen used for *H. pylori* eradication

Lineage	Regimen	Dosage	Duration	Caution	References
Antibiotic susceptibility test if available					
First line Grade of recommendation: Strong	PPI triple Therapy (PAC)	• PPI^a^ bid • Amoxicillin 1000 mg bid • Clarithromycin 500 mg bid	14 days	Should be administered with caution in some regions of Indonesia with high Clarithromycin resistance (≥ 15%)^d^ or personal history of macrolide exposure	[7, 81]
Alternative regimen therapy Grade of recommendation: Strong	Concomitant non-bismuth quadruple therapy (PAMC)	• PPI^a^ bid • Amoxicillin 1000 mg bid • Metronidazole^b^ 500 mg bid (or Nitroimidazole) • Clarithromycin 500 mg bid	14 days	• Can be used when bismuth is not available • Can be the first lineage in areas with high clarithromycin resistance (≥ 15%)^1^, if an antibiotic susceptibility test is not available • Can be used for patients with true penicillin allergy	[1, 5–9, 81]
Alternative regimen therapy Grade of recommendation: Conditional	Bismuth quadruple Therapy (PBMT)	• PPI^a^ bid • Bismuth^c^ qid • Metronidazole^b^ 400 mg qid or 500 mg tid–qid (or Nitroimidazole) • Tetracycline 500 mg qid	14 days	• Can be first lineage in areas with high clarithromycin resistance (≥ 15%)^d^, if antibiotic susceptibility test not available • Can be an alternative rescue if the first line is failure • Currently, bismuth is not available in Indonesia	[1, 5–9]
An antibiotic susceptibility test is strongly recommended					
Alternative rescue Grade of recommendation: Strong	High-dose dual therapy	Rabeprazole 20 mg qid Amoxicillin 750 mg qid	14 days	• Can only be used as an alternative rescue when the first lineage fails and in areas with high resistance to levofloxacin (≥ 15%)^e^ • Should be used in areas with rapid metabolizer population	[5, 6, 8]
Alternative rescue Grade of recommendation: Conditional	Rifabutin-containing therapy (PAR)	PPI^a^ bid Amoxicillin 1000 mg bid Rifabutin 150 mg bid or 300 mg qd	10 days	• Can only be used as an alternative rescue as the third or fourth line of treatment	[5, 6, 8]

^a^The dose depends on the PPI used. The standard doses are dexlansoprazole, 30 mg; esomeprazole, 20 mg; lansoprazole, 30 mg; omeprazole, 20 mg; pantoprazole, 40 mg; and rabeprazole, 20 mg, although a double dose is sometimes used for dexlansoprazole, esomeprazole, omeprazole, and rabeprazole to increase the success of eradication

^b^Metronidazole may be substituted by tinidazole

^c^The dose depends on the formulation used. Examples include bismuth subsalicylate (262 mg), two tablets, colloidal bismuth subcitrate (120 mg), one tablet

^d^An area with high resistance to clarithromycin (≥ 15%) is Bali

^e^Areas with high resistance to levofloxacin (≥ 15%) are Bali, Java, Kalimantan, Papua, Sulawesi, Sumatra, and Timor

##### Statement 25

An alternative treatment regimen should be used based on local antibiotic resistance data or a clinical *H. pylori* antibiotic resistance test (if available). The alternative treatment regimen that can be used is concomitant non-bismuth quadruple therapy (PAMC) and bismuth quadruple therapy (PBMT). However, since bismuth is not yet available in Indonesia, PAMC is the rational choice. Hence, this consensus recommends the Indonesian government to provide bismuth for this purpose.

**Grade of recommendation:** Conditional.

**Level of evidence:** High.

**Rationale:**

Several guidelines recommend PAMC and PBMT as the first line treatment regimen for *H. pylori* eradication [1, 5–9, 81]. However, since metronidazole resistance was high in Indonesia, PPI triple therapy remains the first choice considering that it remains effective in the general Indonesian population with cautious use in some regions of Indonesia with high clarithromycin resistance (≥ 15%) (e.g., Bali) or personal history of macrolide exposure [81, 83] (Table 5; Fig. 5).

##### Statement 26

An antibiotic susceptibility test must be conducted in the patients that not responding (failed) to any treatment regimen. Either an E-test or agar dilution method can be used. If antibiotic resistance is detected, high-dose dual therapy or rifabutin-containing therapy (PAR) can be considered as the alternative rescue regimen after the failed therapy.

**Grade of recommendation:** Strong.

**Level of evidence:** High.

**Rationale:**

An antibiotic susceptibility test must be conducted in patients who failed to respond to any treatment regimen because the antibiotic resistance might have occurred during the previous treatment [73]. Clinicians should not easily re-administer any of the antibiotics against which *H. pylori* has most likely developed resistance in the event of *H. pylori* treatment failure [84]. The E-test or agar dilution method should be performed before deciding the next regiment therapy. A study (conducted using *H. pylori* Indonesian strains) found that despite occasional inconsistencies between these two methods, the E-test shows satisfactory agreement for levofloxacin, metronidazole, tetracycline, and clarithromycin, though additional confirmation for amoxicillin may be required [85]. Subsequently, if antibiotic resistance is confirmed, high-dose dual therapy or PAR can be considered as an alternative rescue regimen after the failure of therapy [5, 6, 8].

##### Statement 27

Dose adjustment of *H. pylori* eradication therapy should be considered when administering drugs affected by CYP2C19 in Indonesia.

**Grade of recommendation:** Conditional.

**Level of evidence:** High.

**Rationale:**

CYP2C19 is an enzyme involved in the metabolism of a variety of medications, including PPIs [86]. The polymorphism of CYP2C19 can alter the therapeutic efficacy of medicines. Intermediate and rapid metabolizers were found to be the most common in Indonesia. This condition may be different between ethnicities [87]. Because CYP2C19 can alter the clinical efficacy of *H. pylori* eradication therapy medications, dose adjustments should be considered in Indonesia when using PPI-based therapy [87].

##### Statement 28

In Indonesia, the amoxicillin, tetracycline, rifabutin, sitafloxacin, and furazolidone resistance rates are remain low, whereas levofloxacin, rifaximin, and metronidazole resistance are high.

**Grade of recommendation:** Not applicable.

**Level of evidence:** High.

**Rationale:**

The resistance of *H. pylori* to amoxicillin remains very low; therefore, amoxicillin should be considered as a first-line treatment. Additionally, resistance to other antimicrobials, sitafloxacin, rifabutin, furazolidone, and tetracycline is currently low. However, resistance to levofloxacin and metronidazole is considerably high in Indonesia (generally > 30%) [66, 81]. There was no evidence stating that the percentage of resistance to these antibiotics was related to an increased use of these antibiotics [10, 88]. Future study to determine new regimen therapy or new drug discovery should performed with the collaboration of the government, clinicians, academicians, and researchers.

##### Statement 29

Bismuth quadruple therapy as solution in amoxicillin allergy and would depend on the local pattern of susceptibility and the patient’s drug allergy status.

**Grade of recommendation:** Strong.

**Level of evidence:** Moderate.

**Rationale:**

The definition of amoxicillin allergy includes the following criteria: (1) History of allergic reactions such as fever, rash, itchy skin, and anaphylactic shock after oral, intramuscular, or intravenous administration of penicillin. (2) A positive skin test. Bismuth quadruple therapy containing of a PPI, tetracycline, metronidazole, and bismuth (or another nitroimidazole) for 10–14 days is the recommended first-line treatment. Bismuth quadruple therapy is particularly engaging to patients who have been previously exposed to macrolides or who are allergic to penicillin. Alternative options depend on local susceptibility patterns (see Table 5 for alternative treatment options) [7].

##### Statement 30

Potassium-competitive acid blocker (i.e., vonoprazan and tegoprazan)-based therapy is a new promising alternative PPI-based treatment.

**Grade of recommendation:** Not applicable.

**Level of evidence:** Moderate.

**Rationale:**

Compared to conventional PPIs, potassium-competitive acid blockers (e.g., vonoprazan and tegoprazan) are more efficient as antisecretory drugs because several reasons as follows: (1) more rapid onset of action, (2) reduced reported antisecretory fluctuations, (3) higher safety and (4) better tolerability. A 2017 meta-analysis showed multiple benefits of vonoprazan-based triple regimen compared to conventional PPI-containing regimen [89]. Further studies also suggested that vonoprazan could be used as a first- or second-line regimen for *H. pylori* eradication [90]. In addition, other studies showed that not only as triple therapy but vonoprazan double therapy (7-day vonoprazan and low-dose amoxicillin) was also acceptable for *H. pylori* eradication [91]. Further study regarding the effectivity of vonoprazan in Indonesia is recommended.

## Data Availability

Not applicable.

## Abbreviations

CLO: *Campylobacter*-Like organisms
EGC: Early gastric cancer
EGD: Esophago-gastro-duodenoscopy
EPS: Epigastric pain syndrome
FD: Functional dyspepsia
HDL: High-density lipoprotein
IDA: Iron deficiency anemia
ITP: Immune thrombocytopenic purpura
MALT: Mucosa-associated lymphoid tissue
NNT: Number needed to treat
PDS: Postprandial distress syndrome
PPE: Personal protective equipment
PUD: Peptic ulcer disease
RUT: Rapid urease test
SAT: Stool antigen test
UBT: Urea breath test
